# Acute Progression of BCR-FGFR1 Induced Murine B-Lympho/Myeloproliferative Disorder Suggests Involvement of Lineages at the Pro-B Cell Stage

**DOI:** 10.1371/journal.pone.0038265

**Published:** 2012-06-06

**Authors:** MingQiang Ren, Josephine A. Tidwell, Suash Sharma, John K. Cowell

**Affiliations:** 1 Georgia Health Sciences University Cancer Center, Georgia Health Sciences University School of Medicine, Augusta, Georgia, United States of America; 2 Department of Pathology, Georgia Health Sciences University School of Medicine, Augusta, Georgia, United States of America; University of Navarra, Center for Applied Medical Research, Spain

## Abstract

Constitutive activation of FGFR1, through rearrangement with various dimerization domains, leads to atypical myeloproliferative disorders where, although T cell lymphoma are common, the BCR-FGFR1 chimeric kinase results in CML-like leukemia. As with the human disease, mouse bone marrow transduction/transplantation with BCR-FGFR1 leads to CML-like myeloproliferation as well as B-cell leukemia/lymphoma. The murine disease described in this report is virtually identical to the human disease in that both showed bi-lineage involvement of myeloid and B-cells, splenomegaly, leukocytosis and bone marrow hypercellularity. A CD19^+^ IgM^−^ CD43^+^ immunophenotype was seen both in primary tumors and two cell lines derived from these tumors. In all primary tumors, subpopulations of these CD19^+^ IgM^−^ CD43^+^ were also either B220^+^ or B220^−^, suggesting a block in differentiation at the pro-B cell stage. The B220^−^ phenotype was retained in one of the cell lines while the other was B220^+^. When the two cell lines were transplanted into syngeneic mice, all animals developed the same B-lymphoblastic leukemia within 2-weeks. Thus, the murine model described here closely mimics the human disease with bilineage myeloid and B-cell leukemia/lymphoma which provides a representative model to investigate therapeutic intervention and a better understanding of the etiology of the disease.

## Introduction

The 8p11 myeloproliferative syndrome (EMS), also known as stem cell leukemia lymphoma (SCLL) syndrome, is a distinct clinico-pathological entity [1] defined by reciprocal chromosome translocations that result in a chimeric protein with constitutive activation of the kinase domain of the fibroblast growth factor receptor-1 (FGFR1). As such, the WHO has now reclassified this entity as a “myeloid and lymphoid neoplasm with FGFR1 abnormalities” [2], [3]. The fusion partner protein provides the oligomerization required for ligand-independent activated FGFR1 signaling, which is essential in pathogenesis [4]. To date, at least 11 FGFR1 fusion partners have been identified, most of which induce a myeloproliferative neoplasm or disorder (MPN/MPD) and lymphadenopathy, usually involving T-lymphoblastic lymphoma/leukemia predominantly of an immature T-cell type. The t(8;22) variant translocation, results in a fusion between BCR (breakpoint cluster region) and FGFR1 which is clinically distinct from the myeloid/T-cell neoplasms of other variant FGFR1 fusions [5], [6], [7], [8], [9], [10], [11]. Leukemias in these patients are clinically more similar to BCR-ABL1 induced CML, suggesting BCR may play a role in this particular pathogenesis.

Currently, only eleven patients with the variant BCR-FGFR1 rearrangement have been described in detail. These patients were usually diagnosed with an atypical CML (aCML) myeloid neoplasm characterized by basophilia, splenomegaly, leukocytosis, fibrosis and a hypercellular marrow, with an increase in immature cells of the granulocyte series creating a left shift in granulopoiesis [5], [6], [7], [8], [9], [10], [11]. In addition to aCML, 5 out of 11 BCR-FGFR1 patients also showed varying numbers of cells expressing B-cell markers [6], [7], [9], [11], [12] and two of them presented with a predominantly acute precursor B-lymphoblastic leukemia (preB-ALL) [6], [12]. Only one patient developed both MPD and T-cell lymphoblastic lymphoma [13]. Thus, the t(8;22) appears to be able to induce a simultaneous myeloid and B lymphoid MPD.

In a murine model of BCR-FGFR1, Roumiantsev et al. [14] demonstrated that the BCR-FGFR1 fusion kinase induced a CML-like neoplasm devoid of T-cell lymphomas. The involvement of B-cell proliferation in this model, however, was not reported, which is inconsistent with the human disease. We recently described a transduction-transplantation model of ZNF198-FGFR1-induced SCLL, where the phenotype was almost identical to the human disease [15]. We have now extended this approach to include a model of BCR-FGFR1 induced leukemia were, in contrast to the previous report, mice rapidly developed SCLL syndrome which was similar to the human disease as evidenced by hepatosplenomegaly, fibrosis and leukocytosis with progenitor, myeloid and B-cell neoplasms in the bone marrow and spleen that were transplantable and tumorigenic in secondary recipient mice. These observations suggest that the BCR-FGFR1 fusion kinase induces murine leukemia which is consistent with the human disease and, in agreement with Murati and colleagues [9], suggests that BCR-FGFR1 represents a distinct FGFR1 fusion-induced hematopoietic neoplasm. During the course of these experiments we also developed two cell lines from leukemic mice that are immunophenotypically consistent with a pro-B cell progenitor.

## Materials and Methods

### Creation of the BCR-FGFR1 Mouse Model

The BCR-FGFR1 fusion cDNA construct, MSCV-GFP-BCR-FGFR1, was a kind gift from Dr. Richard Van Etten [14]. The original BCR-FGFR1 cDNA [7] was subcloned into the *EcoRI*-site of a murine stem cell virus (MSCV) vector that contains an internal ribosomal entry site (IRES) and the green fluorescent protein (GFP) [14]. This insert was then subcloned into the *EcoR*I/*NotI* site of the MIEG3 vector which provides enhanced GFP (EGFP) expression (provided by Dr Wen Tao) [16]. Bone marrow (BM) from donor mice was transduced with either MIEG3-BCR-FGFR1 or MIEG3 in vitro as described previously [15]. Briefly, BALB/cAnNTac (Taconic Farms), 6- to 8-week-old mice were used throughout. For BM cell transduction, male donor mice were first treated with 5-fluorouracil (150 mg/kg) intraperitoneal injection, 2 days before the collection of BM cells. Recovered cells were then incubated in IL2, IL3, and stem cell factor (final concentration 100 ng/ml for each) for 24 hours before infection. After prestimulation, 4 rounds of infection at 12-hour intervals were performed with the retroviral supernatant (25% vol/vol) in the presence of 10 g/ml polybrene, using the same media and cytokines as described above. The infected BM cells (1.5×10^6^ cells per mouse) were then injected into lethally or sublethally irradiated (900 or 600 cGy respectively) recipient mice via tail vein injection. All animal experiments were carried out under protocol was approved by the Institutional Animal Care and Use Committee of the Georgia Health Sciences University.

### Histopathology

Peripheral blood smear and tissue fixation and staining were as described previously [15].

### Flow Cytometric Analysis

Antibody details are provided in Table S1 and staining methods were as described previously [15].

### Molecular Analyses

Total RNA was prepared using standard TRIzol (Invitrogen) isolation and 5 ug of total RNA was retrotranscribed with SuperScript first-strand synthesis III (Invitrogen) and 1 µl cDNA, relative to 250 ng RNA, was amplified by PCR. Primers used to identify the BCR-FGFR1 breakpoint were; Forward – TGCCCTACATTGATGACTCG; Reverse – TTGGAGGCCAGATACTCCAT.

### Cytogenetic and Comparative Genomic Hybridization (CGH) Analyses

Chromosomes were analyzed using Spectral Karyotyping (SKY) as described previously [17]. Genomic DNA was extracted from BBC1 and BBC2 cells using standard methods and array CGH was performed using Agilent’s SurePrint G3 Mouse CGH Microarry 4×180 K (G4839A, Agilent Technologies). Labeling and hybridization of genomic DNA was performed according the manufacturer’s instructions. DNA from a normal female Balb/c mouse spleen was used as reference DNA during array-CGH. After post-hybridization washes, the arrays were scanned and the spot intensities were measured using “Feature Extraction Software” (version 10.7, Agilent Technologies). The raw array data was acquired using the feature Extraction Sofware and subsequently analyzed by CGHWeb (http://compbio.med.harvard.edu/CGHweb) using circular binary segmentation (CBS, alpha = 0.05) algorithms.

## Results

### BCR-FGFR1 Induces Both a CML-like Myeloproliferative Disease and B-cell Leukemia/lymphoma in a Mouse Model

**Figure 1:**
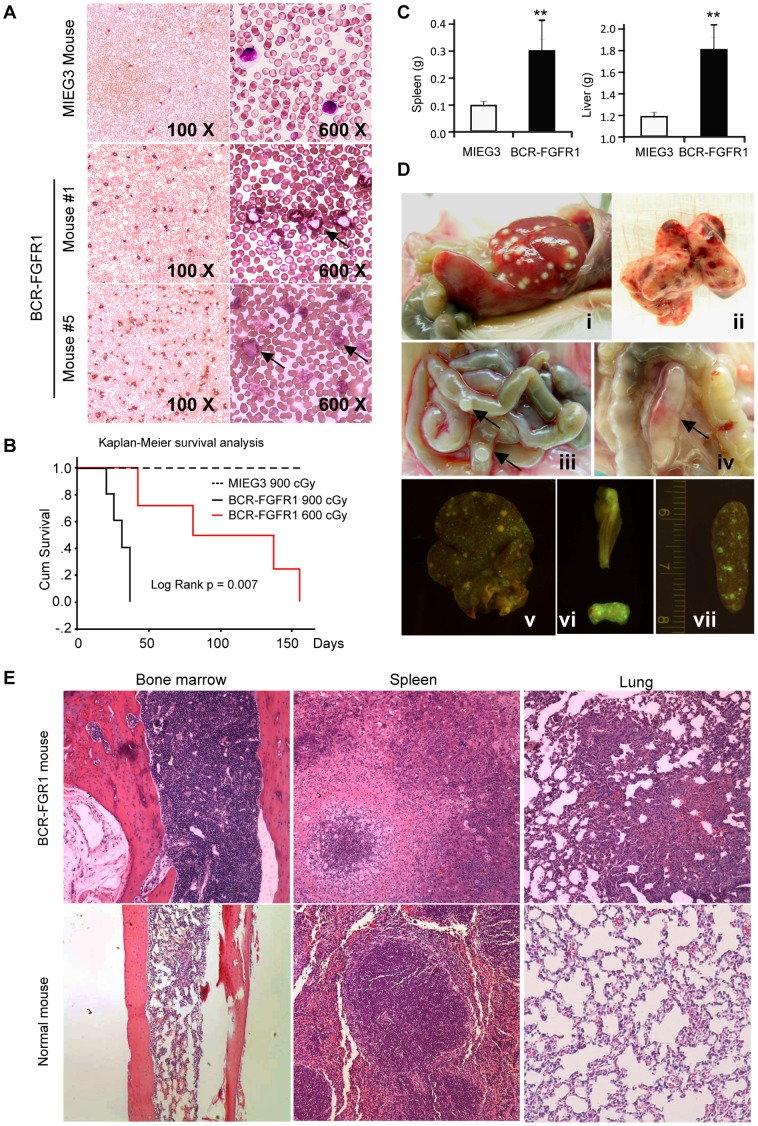
A mouse model of BCR-FGFR1 induced leukemia is consistent with the human disease. (A) May-Grünwald-Giemsa staining of peripheral blood showing an increase of white blood cells and the presence of blasts with a more mature myeloid chronic-like phenotype from mouse #1 (above) and a more blast-like phenotype from mouse #5 (below) compared with BM from mice reconstituted with the MIEG3 vector. (B) Survival curves for mice receiving either 900 centiGray or 600 centiGray shows a longer latency period for the lower dose and longer survival compared with MIEG3 controls. (C) The average spleen and liver weight of BCR-FGFR1 recipients (n = 8) were significantly increased compared to MIEG3 mice (n = 5), (** p<0.01). (D) BCR-FGFR1 induced leukemia characterized by (i) hepatosplenomegaly and visible splenic fibrosis, (ii) pulmonary infiltration, enlarged Peyer’s patches (iii, arrows) and a mesenteric lymph node (iv, arrow). Molecular imaging of GFP defined tumor cell populations in the liver (v), Peyer’s patches (vi) and spleen (vii). (D) H&E stained BCR-FGFR1 recipient tissues shows hypercellular bone marrow with a predominant increase in myeloid and megakaryocytic lineages, spleen with extramedullary myeloid proliferation including dysplastic megakaryocytes (middle) and infiltrating myeloid cells in the lung which is consistent with D (ii). Tissues from control animals are shown below for comparison.

Donor bone marrow (BM) from three individual Balb/c founder mice were transduced with the BCR-FGFR1-containing MIEG3 viral vector which was then transplanted into 5 lethally irradiated (900 cGy) recipient mice. To investigate the effect of irradiation dose on disease development, we also independently infected BM cells from an additional 5 donor mice with BCR-FGFR1 retro-supernatant. These infected BM cells were transplanted into 5 sublethally irradiated (600 cGy) recipients. One mouse in this group died from irradiation shortly after transplantation and no obvious disease development was noted. Approximately 2-weeks following the primary transplantation, analysis of peripheral blood samples from these animals showed that the WBC count in the peripheral blood began to reconstitute and increase, suggesting successful engraftment. By 3 to 4 weeks post-transplantation the WBC count was remarkably increased in all lethally irradiated recipients (Figure 1A). Abnormal leukocytes with ring-shaped nuclei and blast cells were frequently seen in this cohort of mice (Figure 1A), indicative of the development of leukemia. The increase of WBC count or abnormal leukocytes were only seen after ∼2 months post-transplantation in sublethally irradiated recipients. The median survival time in the lethally irradiated cohort (n = 5) was 30±6.5 days post-transplantation, while the 600 cGy cohort (n = 4) it was 83±52.5 days (Figure 1B). Except for this longer latency period, we did not observe any differences between lethally and sublethally irradiated recipients based on their immunophenotypes (see below). Five mice transduced and transplanted with the empty MIEG3 vector did not develop disease over a one year observational period.

Pathological analysis showed that all of the diseased mice showed significantly enlarged spleens (p = 0.006) and livers (p = 0.0004) compared with normal mice (Figure 1C and D). Enlarged Peyer’s patches were observed in 8 out of 9 recipients (Figure 1D) and enlarged mesenteric lymph nodes were seen in about 40% mice (Figure 1D). Peripheral lymph nodes were infrequently enlarged. Molecular imaging of the liver, spleen and femur and a fragment of intestine showed GFP positive cells distributed throughout these organs, indicating that they were derived from transformed cells from the donor mice (Figure 1D, bottom). Furthermore, histopathological analyses of the primary transplanted mice showed that the disease was accompanied by hyper-cellularity in the bone morrow from the sternum, and leukocyte infiltration into multiple organs, such as the lung (Figure 1E), liver and heart (data not shown).

### B-cell Leukemia in BCR-FGFR1 Disease Suggests a Block at the Pro-B Stage

**Figure 2:**
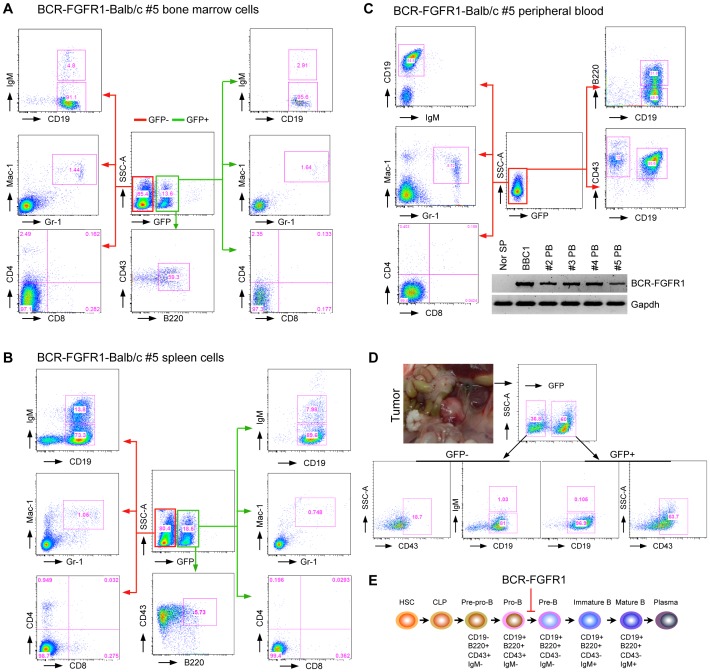
BCR-FGFR1 simultaneously induces B-cell leukemia/lymphoma and myeloid proliferative disorder. Representative flow cytometric analysis of bone marrow (A), spleen (B) and peripheral blood (C) cells from primary transplanted mouse #5 with BCR-FGFR1-induced disease shows that the majority of B-cells are arrested at pro-B stage with a CD19^+^IgM^−^ immunophenotype in both GFP positive and negative cells. The presence of GFP^+^Mac-1^+^Gr-1^+^ populations, as well as high levels of GFP^−^Mac-1^+^Gr-1^+^ cells, was also seen. Very few T-lineage (CD4^+^ and/or CD8^+^) cells were present in the leukemic spleen. GFP expression was lost in the peripheral blood from the majority of primary recipients, where the BCR-FGFR1 fusion transcript can be detected by RT-PCR (C, below). D) Most of the primary transplanted mice showed enlarged lymph nodes and/or tumors in their abdomens. Representative flow cytometric analysis of the tumor cells shows the predominantly pro-B lymphoma immunophenotype, CD19^+^IgM^−^. E) Schematic diagram showing the progression of B-cell development from hematopoietic stem cells (HSC) and common lymphoid progenitors (CLP) into mature B cells and plasma cells. The immunophenotype of the B-lineage cells carrying the BCR-FGFR1 fusion kinase suggests an arrest at the pro-B stage of development as indicated.

Expression of EGFP in the BCR-FGFR1 transformed leukemic cells conveniently permitted flow cytometric analysis of cells carrying the fusion kinase. Premature death of one founder mouse precluded conclusive flow analysis in this case, but the remaining 8 were used in subsequent analyses. Flow cytometric analysis using lineage-specific markers was performed in all the available primary recipients. An analysis of GFP^+^ splenocytes using B cell markers identified populations that were IgM^−^CD19^+^CD43^+^B220^+^ (Figure 2A). GFP^+^ myeloid cells in the spleen showed co-expression of Mac-1 and Gr-1 (Figure 2A) and, in all but one mouse, were found at lower frequencies than the GFP^+^ B cells. As an exception, the majority of GFP^+^ cells in mouse #1 and #2 were Mac-1^+^Gr-1^+^ (Figure S1A) suggesting a predominantly myeloid disease. Since early B-cell development is mainly located in the bone marrow, we also analysed the bone marrow compared with the peripheral blood cells from all the disease mice, which showed a similar phenotype (Figure 2B and C). During this analysis, we noticed that peripheral blood from several primary recipients had lost GFP expression, even though bone marrow cells in these animals contained GFP^+^ cells, and RT-PCR analysis demonstrated that these GFP^−^ cells carried the BCR-FGFR1 fusion gene (Figure 2C). In all the primary recipients, very few (1%) splenocytes showed a GFP^+^CD4^+^CD8^+^ phenotype excluding the possibility of T-cell disease. Most of the primary transplanted mice showed enlarged lymph nodes and/or tumors in their abdomens. These tumor cells were GFP^+^ IgM^−^ CD19^+^CD43^+^ (Figure 2D) which is consistent with a pro-B cell phenotype (Figure 2D). During these analyses we noticed that the GFP^+^ cells only accounted for <20% of whole cell population, suggesting that most tumor cells had lost GFP expression. Consistent with this suggestion, the majority of GFP negative cells shared the same abnormal immunophenotype with GFP positive cells, i.e. IgM^−^CD19^+^CD43^+^ (Figures 2 and S1), Gr1^+^Mac1^+^ B220^+^ or Gr1^+^Mac1^+^CD19^+^ (Figure S1). Furthermore, the BBC1 cell line derived from one primary recipient is GFP negative and expresses the BCR-FGFR1 fusion protein (Figure 2A and 4A). Overall, therefore, BCR-FGFR1 induced both myeloid leukemia and B-cell leukemia/lymphoma blocked at the pro-B stage in most mice and there was no evidence of T-cell disease.

### Murine Leukemia/lymphoma Induced by BCR-FGFR1 is Transplantable

**Figure 3:**
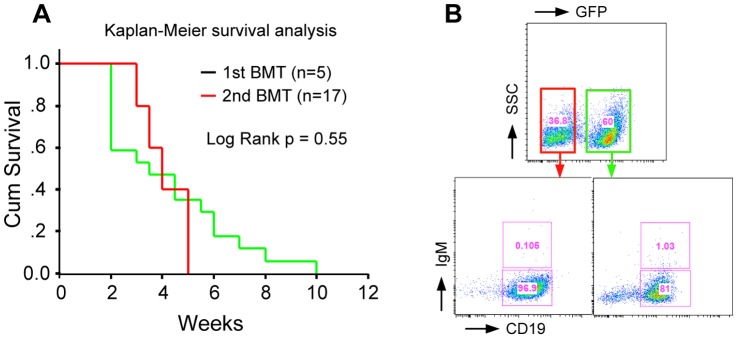
B-cell leukemia/lymphoma induced by BCR-FGFR1 is transplantable. A) Kaplan-Meier survival analysis of the second generation of BCR-FGFR1 mice, which were transplanted with BM cells (0.6–1.5×10^6^ cell per mouse) from lethally irradiated primary recipients. B) Representative flow analysis shows that lymphoma/tumor cells from a second serial transplanted mouse are differentially blocked at the pro-B stage.

To determine transplantability of these lymphomas, BM cells from the 5 lethally irradiated mice were each transplanted into 3–5 secondary recipients that had been sublethally irradiated (600 Gy). Depending on the number of cells obtained from the primary transplanted mice, 0.6–1.5×10^6^ cells were transplanted and leukemias developed within 2–10 weeks suggesting fully transformed leukemic stem cells were present in the BM. The latency period in the secondary transplanted mice was similar to that seen in the primary recipients and the median survival time was ∼ 30 days (Figure 3A). The immunophenotype of the secondary transplanted mice was also similar to that in the primary mice (Figure 3B). Because all secondary transplanted mice simultaneously developed transplantable myeloid and B-lineage disease suggests that the disease initiates from progenitor cells or stem cells.

### BCR-FGFR1 Derived Cell Lines with Pro-B Progenitor Immunophenotypes

**Figure 4:**
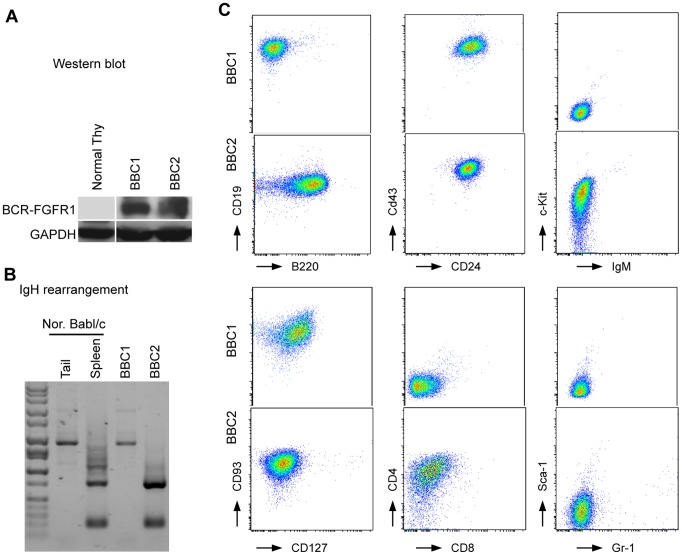
BCR-FGFR1 derived cell lines are consistent with an pro-B cell phenotype. (A) Western blot analysis shows the presence of the 120 kD BCR-FGFR1 fusion protein in BBC1 and BBC2 cells compared with normal thymocytes. (B) Genomic PCR analysis of IgH rearrangement showing the germline configuration in the tail and polyclonal rearrangements in the spleen from normal mice. (C) Flow analysis of BBC1 and BBC2 cell lines demonstrates an immature B-cell immunophenotype.

Cells from the both bone marrow and spleen of leukemic mice were routinely cultured in standard growth medium supplemented with 5% FBS. In two cases (#5 and #7), progressively growing cell lines were established from bone marrow samples. These cell lines are referred to as BBC (BCR-FGFR1 Bone marrow Cells) 1 and 2 and have been grown in suspension for over 50 passages in vitro under normal growth conditions without addition of growth factors. RT-PCR analysis across the breakpoint demonstrated the presence of the BCR-FGFR1 fusion transcript in both cases (not shown) and western blot analysis also confirmed that they both express the BCR-FGFR1 fusion protein (Figure 4A). RT-PCR analysis showed BBC1 cells express TdT, RAG1, EBF-1 and Pax-5 suggesting an immature B cell lineage (data not shown). Analysis of the immunoglobulin rearrangements in these cells demonstrated a germline configuration in the BBC1 cells, whereas BBC2 cells showed oligoclonal rearrangements (Figure 4B). FACS analysis of BBC1 demonstrated a pro-B immunophenotype (c-Kit^−^CD19^+^CD24^+^CD43^+^IgM^−^CD127^+^CD93^+^), albeit B220^−^ (Figure 4C). A sub-population of CD19^+^B220^−^ cells was also seen in the primary donor that gave rise to these cells (Figure 2B), which was also observed in 2 other founder mice (Figure S1). The BBC2 cell line showed an immunophenotype which was CD19^+^CD24^+^cKit^+^ IgM^−^CD43^+^CD93^+^ but in this case was B220^+^ supporting a pro-B cell origin. These cells also expressed CD4 (Figure 4C), demonstrating biphenotypic characteristics seen in cells from SCLL patients.

### BBC Cells Rapidly Induce Disease in Mice

When BBC1 or BBC2 cells were transplanted, individually into normal BALB/c mice, aggressive leukemia/lymphoma and splenomegaly developed in all cases after only 14 days (Figure S2). In addition, leukocytosis (Figure S2B) and infiltration into various organs was observed (Figure S2C). Histopathologically, spleens show intense extramedullary leukocyte proliferation, including maturing but mostly numerous precursor and blast forms, together with dysplastic megakaryocytes (Figure S2D).

### Cytogenetic Analysis of BBC Cell Lines

**Figure 5:**
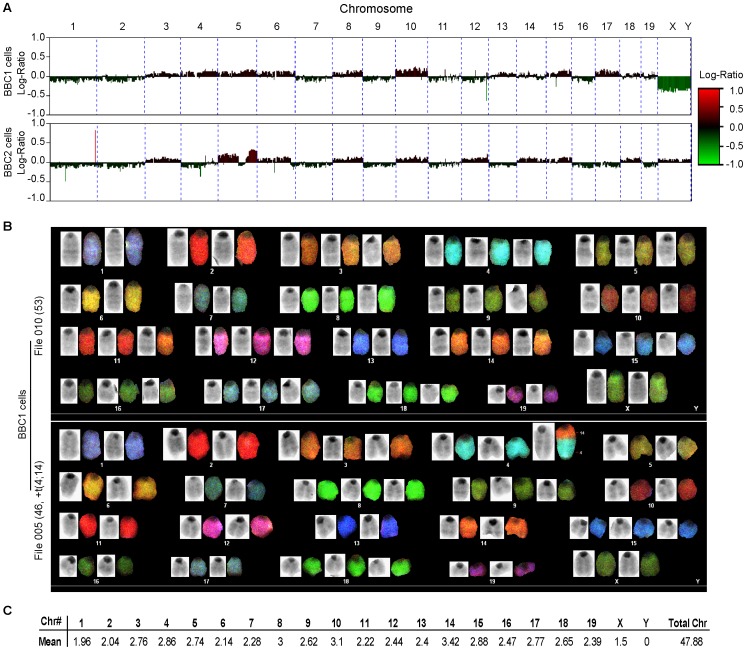
Karyotypic analysis of B-lymphoma cell lines. CGH analysis (A) of BBC1 (above) and BBC2 (below) show distinct chromosome changes in the two cell lines. SKY analysis of BBC1 cells (B) demonstrates extensive aneuploidy and, in one metaphase, a t(4;14) non-reciprocal chromosome translocation.

Analysis of the chromosomes from the BBC1 cell line at an early stage (passage 5–10) using Spectral Karyotyping (SKY), demonstrated highly unstable karyotypes with multiple trisomies and a large variation in the chromosome composition between individual cells. Examples in Figure 5 show different clones within the parent cell population. The only chromosome translocation identified was a t(4;14) which was only seen in 3/51 metaphases analyzed and to our knowledge has not been reported previously. Comparative genome hybridization (CGH) analysis at a later passage (>50) demonstrated even further evolution of the chromosome content (Figure 5) with many whole chromosome losses and gains. CGH analysis of BBC2 similarly showed extensive chromosome aneuploidy where some of the changes were in common with BBC1 cells, although others were specific to BBC2. Common changes in the two cells lines were; −1, −2, +3, +5, +6, +8, −9, +10, +15 and −16. The high level of aneuploidy in these B-lymphoma cell lines is in contrast with those seen in the T-lymphoma cell line carrying the ZNF198-FGFR1 chimeric kinase [15] where only minimal aneuploidy was noted with a deletion of the TCRA locus on chromosome 14. This deletion was not seen in the BBC1/2 cell lines.

## Discussion

The EMS/SCLL-related leukemia/lymphoma syndromes represent a subgroup of cancers for which the prognosis is dismal, even despite chemotherapy and BMT [4]. Despite the clear involvement of the FGFR1 fusion kinases, still relatively little is known about the etiology of these diseases and the distinct phenotypes seen in patients with the rarer FGFR1 rearrangements provides an opportunity to dissect their subtle differences. Since the biphenotypic nature of the disease implies a multipotent cell origin, SCLL provides an opportunity to gain a better understanding of the fundamental genetic changes that occur during development and progression of this disease. We have now shown the involvement of a pro-B cell in a mouse model of BCR-FGFR1 SCLL, which may guide future studies in the analysis of the human disease. As a result, more customized approaches or possible alternative therapies may be suggested based on a more detailed analysis of these tumor cells.

To date, BCR-FGFR1 SCLL patients show different manifestations of the disease with some showing B-cell lymphomas as well as myeloid disease. Although it is a common finding that leukemias demonstrate lineage infidelity for their immunophenotypes, the disease in the mice described in this report clearly show characteristics consistent with the human disease. In both species, bi-lineage involvement of myeloid and B-cells, splenomegaly, leukocytosis and bone marrow hypercellularity are common. In addition, during our analysis, all founder mice had a chronic-like, or a B-cell blast-like phase, although none progressed to AML. In a previous report [14] it was suggested that BCR-FGFR1 produced myeloid disease, with no evidence of B-cell lymphoma. It is difficult to determine why the disease is apparently different in what was essentially the same bone marrow transduction and transplantation experimental approach. Although we used an MIEG3-BCR-FGFR1 construct, the only difference is the replacement of traditional GFP with an enhanced GFP reporter in this vector. Both constructs are based on the murine stem cell virus. It has been reported that transformation of bone marrow cells with the BCR-ABL1 fusion kinase induces different lineage diseases depending on whether the donor mice were treated with 5-FU before collection of the BM cells, as well as whether cytokines were used during infection of the BM cells [18]. In our procedure, the donor mice were treated with 5-FU and the BM cells and cultured with the same cytokines as in the previous report [14] We used 150 mg/kg 5-FU to treat the donor mice compared with 200 mg/kg [14]. We do not believe that this minor difference can drive the developed of pro-B cell leukemia and lymphoma. Moreover, we have shown that ZNF198-FGFR1-transduced bone marrow cells developed MPD together exclusively with T-cell lymphoma [15]. Using exactly the same approach, we now show that BCR-FGFR1 transduced bone marrow developed CML-like disease together with only B-cell leukemia/lymphoma in two independent series of experiments. These observations suggest that it is perhaps the specific chimeric FGFR1 fusion kinase that drives the disease development into different lineages rather than the experimental approach or individual mouse strain. In the previous study [14], however, it appears that the absence of B-cell lymphomas was based on an analysis using only the B220 marker, which was negative on the GFP^+^ cells. Ordinarily this would exclude a B-cell lineage, although in our study we also analysed other early B-cell markers where, in mouse #5 for example, a predominantly B220^−^ B-cell lymphoma was seen. In most other mice there were subpopulations of B220^−^ as well as B220^+^ B-cells. Two of the mice in this study (#1 and #2), however, also showed a predominantly (B220^−^) myeloid disease (Figure S1), although there were sub-populations of CD43^+^ and CD19^+^ cells. Most of the other 7 mice showed B220^+^ and B220^−^ populations that were CD19^+^ and CD43^+^. Thus, since CD19 and CD43 markers were not assayed in the previous study [14], it is possible that the lymphomas in this study were, in fact, pro-B cells similar to the BBC1 cells.

The murine stem cell virus-based vector with bi-cistronic expression of GFP has become more frequently used to generate murine bone marrow transduced and transplantation models, as well as human CD34+ xenotransplantation mouse models [19], [20]. We noticed, however, that not all transformed cells simultaneously expressed chimeric FGFR1 and GFP in this study as well as in either the ZMYM2-FGFR1 model [21] or a CEP110-FGFR1 mouse model (Ren et al, manuscript under preparation). We now show that this is probably due to inactivation of GFP expression in the transformed cells during their evolution. Careful analysis of the GFP negative cells demonstrated that they showed the same immunophenotype as the GFP+ cells in the same animal and that they expressed the BCR-FGFR1 chimeric protein.

In summary we have established a representative murine model for BCR-FGFR1 SCLL that has extensive similarities to the human disease and so provides a model that can be used to investigate novel therapeutics for an aggressive disease that is currently refractory to therapy. Although rare, the mechanism by which BCR-FGFR1 SCLL progresses, also represents a model for cancer with clonal evolution that is likely shared by other MPDs and other common cancers. Furthermore, in this model, the molecular pathways that contribute to the transformation to B-ALL, rather than AML, provides an opportunity to investigate the etiology of disease progression in SCLL patients.

## Supporting Information

Figure S1 **BCR-FGFR1 induced mouse developed myeloid or pro-B leukemia.** Flow cytometric analysis of bone marrow (BM) and spleen (SP) cells from mouse #1 (A and B respectively) and mouse #2 (C and D respectively) from the 5 primary lethally irradiated recipients shows a high percentage of Gr1^+^Mac1^+^ cells or/and pro-B leukemia, but fewer cells express CD4 or CD8. These phenotypic analyses are compared to BM (E) and spleen (F) samples from mice which were reconstituted with cells carrying the empty MIEG3 vector.
(TIF)

Figure S2 **BBC1 recipient mice develop leukemia/lymphoma.** (A) Spleen weight of BBC1 recipients were increased compared to normal mice of the same age, (** p = 0.002). (B) May-Grünwald-Giemsa staining of peripheral blood showing dysplastic leukocytosis and the presence of blasts (C) BBC1 transplanted mice with hind leg paralysis, inflamed inguinal lymph nodes, formation of large masses on the lower spinal region or in the gut, accumulation of urine, cerebral hemorrhaging or intracranial bleeding and peritoneal membrane thickening. (D) H&E stained BBC spleen shows involvement of myeloproliferative process with acute leukemic transformation.
(DOCX)

Table S1 **Antibodies used in flow analysis.**
(DOCX)
