# Supplementary figures and images for: Acute Progression of BCR-FGFR1 Induced Murine B-Lympho/Myeloproliferative Disorder Suggests Involvement of Lineages at the Pro-B Cell Stage
Source: PLoS One. 2012 Jun 6;7(6):e38265. doi: 10.1371/journal.pone.0038265 (PMC3368885; doi:10.1371/journal.pone.0038265)

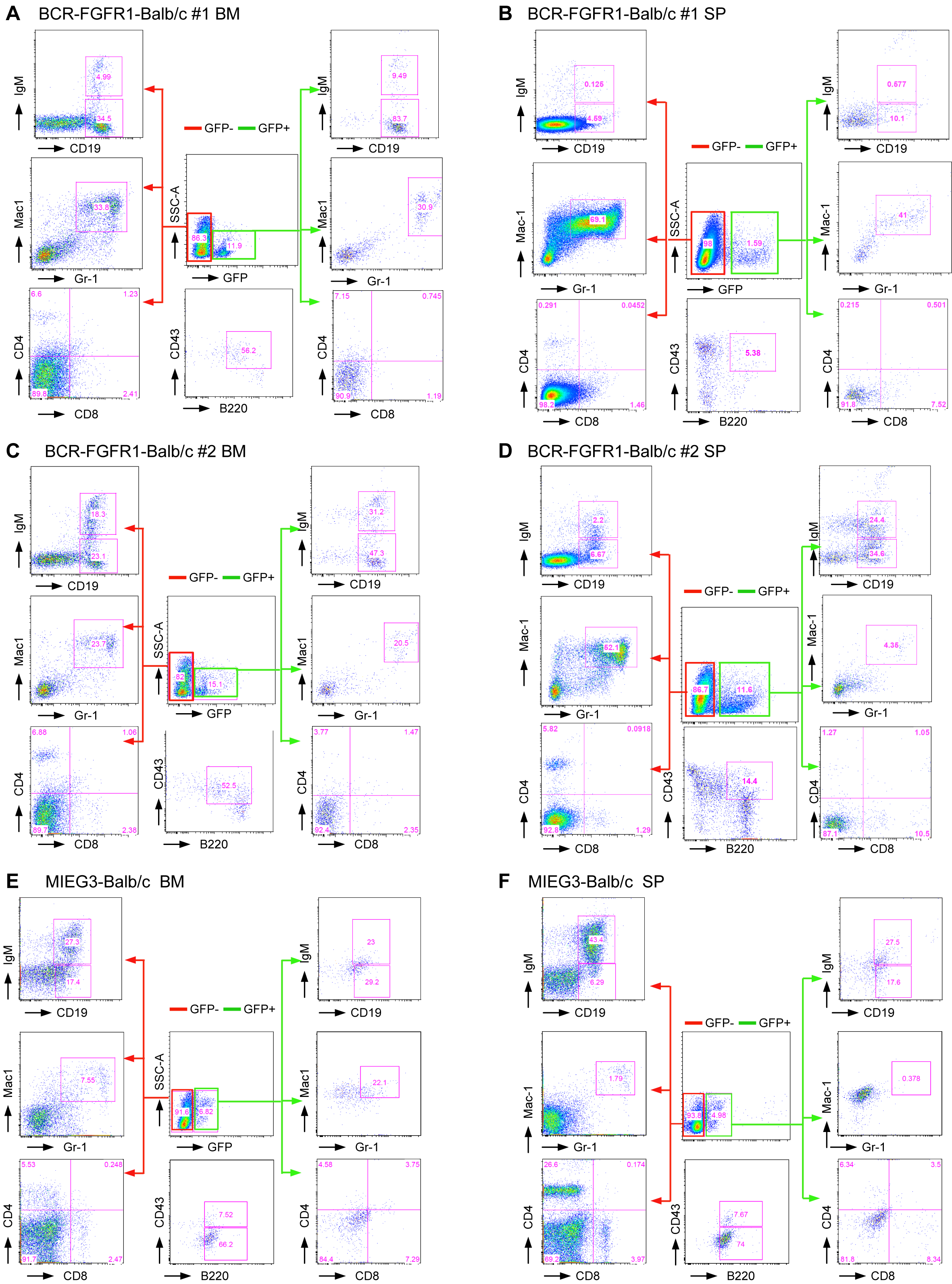

Supplement: Figure S1 — BCR-FGFR1 induced mouse developed myeloid or pro-B leukemia. Flow cytometric analysis of bone marrow (BM) and spleen (SP) cells from mouse #1 (A and B respectively) and mouse #2 (C and D respectively) from the 5 primary lethally irradiated recipients shows a high percentage of Gr1+Mac1+ cells or/and pro-B leukemia, but fewer cells express CD4 or CD8. These phenotypic analyses are compared to BM (E) and spleen (F) samples from mice which were reconstituted with cells carrying the empty MIEG3 vector. (TIF) [file pone.0038265.s001.tif]

**Supplementary figure S2**


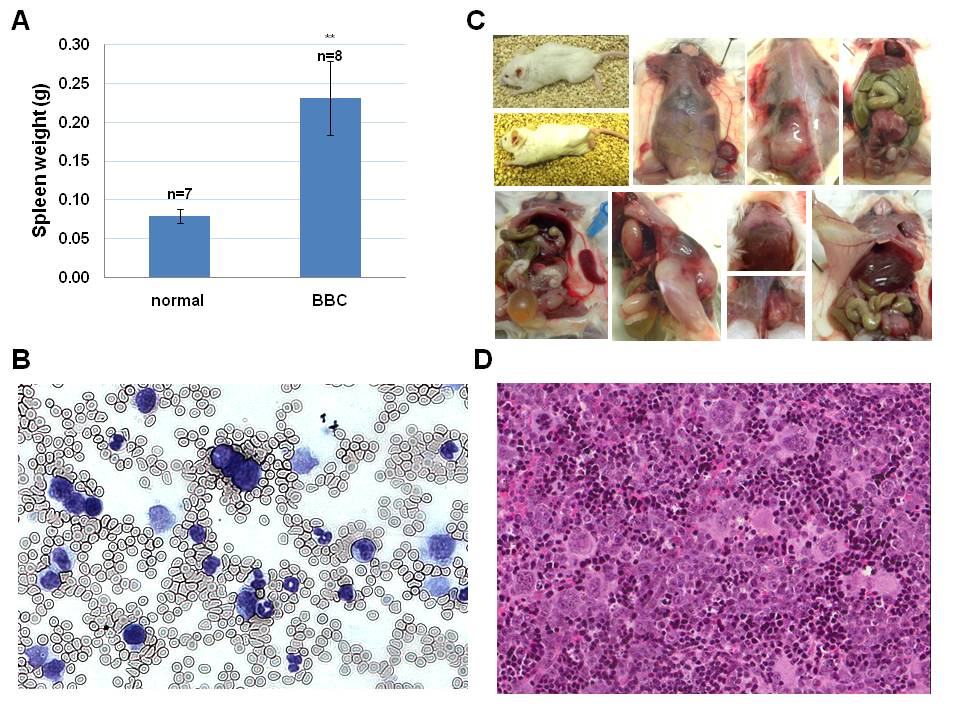

Supplement: Figure S2 — BBC1 recipient mice develop leukemia/lymphoma. (A) Spleen weight of BBC1 recipients were increased compared to normal mice of the same age, (** p = 0.002). (B) May-Grünwald-Giemsa staining of peripheral blood showing dysplastic leukocytosis and the presence of blasts (C) BBC1 transplanted mice with hind leg paralysis, inflamed inguinal lymph nodes, formation of large masses on the lower spinal region or in the gut, accumulation of urine, cerebral hemorrhaging or intracranial bleeding and peritoneal membrane thickening. (D) H&E stained BBC spleen shows involvement of myeloproliferative process with acute leukemic transformation. (DOCX) [file pone.0038265.s002.docx]
