# Supplementary material for: Acute Progression of BCR-FGFR1 Induced Murine B-Lympho/Myeloproliferative Disorder Suggests Involvement of Lineages at the Pro-B Cell Stage
Source: PLoS One. 2012 Jun 6;7(6):e38265. doi: 10.1371/journal.pone.0038265 (PMC3368885; doi:10.1371/journal.pone.0038265)
Supplement: Table S1 — Antibodies used in flow analysis. (DOCX) [file pone.0038265.s003.docx]

**Supplementary Table S1. Antibodies used in flow analysis**

| Progenitor cells | Sca-1-PE-Cy7- (Ly-6A/E) | eBioscience |
| --- | --- | --- |
|  | c-Kit-APC (CD117) | eBioscience |
| T cells | Thy-1-PE | eBioscience |
|  | CD8a-PE-Cy7 | eBioscience |
|  | CD4-AF647 | eBioscience |
| B cells | B220-AF647 (CD45R) | BD Pharmingen |
|  | CD19-PE-Cy7 | BD Pharmingen |
|  | CD43-APC | BD Pharmingen |
|  | IgM-PE | eBioscience |
|  | CD93-PE (AA4.1) | eBioscience |
|  | CD127-PE-Cy5- | eBioscience |
|  | CD24-PE | eBioscience |
| Myeloid cells | Mac-1-APC (Mac-1) | eBioscience |
|  | Gr-1-PE | eBioscience |
